# Influences on reproductive decision-making among forcibly displaced women resettling in high-income countries: a scoping review and thematic analysis

**DOI:** 10.1186/s12939-023-01993-5

**Published:** 2023-09-05

**Authors:** Arielle Donnelly, Greer Lamaro Haintz, Hayley McKenzie, Melissa Graham

**Affiliations:** 1 School of Health and Social Development, Deakin University, Burwood, VIC 3125 Australia; 2 School of Health and Social Development, Deakin University, Geelong, VIC 3220 Australia; 3 Department of Public Health, School of Psychology and Public Health, La Trobe University, Bundoora, VIC 3086 Australia

**Keywords:** Reproductive decision-making, Scoping review, Forced displacement, Women

## Abstract

**Background:**

Forced displacement impacts the health, rights and safety of women, which is further compounded by gender inequality. In particular, this has consequences for forcibly displaced women’s reproductive health once resettled in a new country. To ensure the reproductive health and rights of forcibly displaced women during and after resettlement, there must be careful consideration of their reproductive decision-making taking into account the context and environment of the host country.

**Aim:**

This scoping review aimed to explore the influences on reproductive decision-making among forcibly displaced women resettling in high-income countries.

**Method:**

A scoping review was conducted following the PRISMA-ScR for reporting. EBSCO was used to search databases covering global health, health policy, psychology, sociology, and philosophy for articles published from 1 January 2012 to 27 April 2022. Data extracted from each article included author(s), year of publication, publication type, aims/objectives, study design, sampling method, data collection or eligibility criteria, study population (i.e., sample size and characteristics), migration status, country(ies) of origin, host country(ies), key findings and limitations. Two independent reviewers screened all articles against eligibility criteria using Covidence. Data charting and thematic analysis were performed independently by one reviewer.

**Findings:**

Nineteen articles published between 2013 and 2022 mostly conducted in the United States (36.8%) and Australia (21.1%), with the majority reporting on qualitative findings (68.4%), and women from a wide array of countries and cultures (most commonly African countries) were included. Influences on women’s reproductive decision-making related to the contexts *before displacement, during displacement*, and *after arrival,* with influences on women’s reproductive decision-making identified specific to the context. The influences *before displacement* included *conflict*; *religious beliefs*; *socio-cultural gendered expectations*; and *external control over reproductive autonomy*. *During displacement* influences included *paternalism* and *access to education*. Influences *after arrival* included *pressure, restriction, coercion*; *knowledge and misconceptions*; *patriarchal power dynamics*; and *seeking empowerment*. An adapted socio-ecological model was developed to interpret the findings.

**Conclusion:**

This review highlights the complexity and nuances within forcibly displaced women’s experiences which influence their reproductive decision-making. Further research may review the evidence base to provide guidance for healthcare professionals and health policies aimed at empowering women to make autonomous reproductive decisions; develop training for healthcare professionals to prevent pressure, restriction and coercion of women’s reproductive autonomy; and inform development of policy that takes an intersectional approach to women’s health rights and gender equality.

## Introduction

International human rights enshrine women’s autonomy, equality and reproductive rights with the understanding that reproductive health is an essential part of women’s health. The right to make autonomous reproductive decisions ensures women are empowered to experience the highest standard of health [1]. Forced displacement threatens safety and wellbeing [2], which for women is compounded by gender inequality, often resulting in women’s rights, safety and health being compromised [3]. Despite efforts towards gender equality in high-income countries (HICs) (such as Australia), inequalities (e.g., sexism, racism) continue to impose barriers that prevent migrant women from achieving the highest standard of health [4]. It is important to keep HICs accountable to the 2030 Agenda for Sustainable Development [5], that ensures women’s right to a healthy life, as well as to gender equality through empowerment of women [5]. This scoping review situates women’s reproductive health within gender equality, reproductive rights, and thus, health equity aligned with sustainable development goals. In doing so, it aims to explore the influences on reproductive decision-making among forcibly displaced women resettling in high-income countries.

Good health is necessary to actively participate in society, whereby poor health can create new obstacles including social isolation [6]. Forcibly displaced women experience substantial health risks and consequences related to organised violence and conflict, as well as violence against women (especially sexual violence) before resettlement [7]. Subsequently, many refugee women have trauma related to experiences of conflict and violence, which then continue to impact their health during resettlement [8]. Women’s health may change negatively or positively after arrival in a HIC. More often forcibly displaced women experience declines in mental and physical health [6, 9], which can be influenced by cultural factors (such as language barriers), socio-economic factors (such as secure employment), and personal factors (such as family separation, trauma) [10]. Women also experience barriers to accessing healthcare after arrival in HICs [8]. Cultural differences in HICs impact forcibly displaced women’s health, including experiences of racism from healthcare professionals [11]. These factors also impact women’s reproductive health and access to reproductive healthcare after arrival in HICs [12]. In Australia, refugee women are more likely than Australian-born women to experience poorer maternal and child health outcomes, and less likely to have sufficient information about contraceptive methods [13]. Asylum-seeking women in the United Kingdom begin pregnancy as lower-risk patients (younger and with fewer comorbidities) yet experience poorer reproductive outcomes (miscarriage and higher morbidity) compared to the host population [14]. Moreover, structural barriers (e.g., visa status preventing employment) and symbolic violence (e.g., racism) are commonly experienced by forcibly displaced women during resettlement, which increases women’s risk of experiencing intimate partner violence [15], and reproductive coercion [13].

Reproductive decision-making (RDM) has been defined as, “If and when to have a child or children, the timing, spacing and number of those children; and choices about whether or not to utilise mechanisms for controlling fertility including contraception, assisted reproduction, and abortion” [16] (p.10). Cultural and religious beliefs have been found to influence forcibly displaced women’s RDM [17, 18], for example, forbidding abortion and certain types of contraception [18], and beliefs that women’s fertility is controlled by God [17]. Women in refugee camps have been found to endure gender-based violence (especially sexual violence) more frequently, and several restraints on their reproductive autonomy including restricted access to contraception and abortion [19]. After arrival in a HIC, women may change their preferences about family size by limiting the number of children they have and by utilising available contraceptive methods to reduce financial burden [20].

Overall, the current literature shows forcibly displaced women experience declines in health after arrival in HICs [6, 8–10], poorer reproductive health outcomes compared to the host population [13, 14], and several barriers to access health services [8, 11], including reproductive health services [12], as well as barriers that increase their risk of experiencing reproductive coercion [13, 15]. Despite the existing evidence base, the influences on women’s RDM after arrival are less clear. It is also important that any exploration into the available evidence includes consideration of historical influences before and during women’s displacement because these likely add crucial understanding about the influences on RDM after arrival in HICs. This review focused on the influences on women’s RDM after arrival in HICs and considered influences before and during displacement retrospectively. To this end, this scoping review was undertaken to answer the research question: What influences reproductive decision-making among forcibly displaced women resettling in high-income countries?

## Methods

This scoping review followed the Preferred Reporting Items for Systematic reviews and Meta-Analyses extension for scoping reviews (PRISMA-ScR) (see Table 1 for PRISMA-ScR checklist) [21]. A scoping review was appropriate because it aimed to identify the type and scope of available evidence and critically discuss knowledge gaps, as opposed to a systematic review that focuses on answering a clinically meaningful question [22].

**Table 1:** PRISMA-ScR Checklist

SECTION	ITEM	PRISMA-ScR CHECKLIST ITEM	REPORTED ON PAGE #
TITLE			
Title	1	Identify the report as a scoping review	1
ABSTRACT			
Structured summary	2	Provide a structured summary that includes (as applicable): background, objectives, eligibility criteria, sources of evidence, charting methods, results, and conclusions that relate to the review questions and objectives	2–3
INTRODUCTION			
Rationale	3	Describe the rationale for the review in the context of what is already known. Explain why the review questions/objectives lend themselves to a scoping review approach	6
Objectives	4	Provide an explicit statement of the questions and objectives being addressed with reference to their key elements (e.g., population or participants, concepts, and context) or other relevant key elements used to conceptualize the review questions and/or objectives	6
METHODS			
Protocol and registration	5	Indicate whether a review protocol exists; state if and where it can be accessed (e.g., a Web address); and if available, provide registration information, including the registration number	N/A
Eligibility criteria	6	Specify characteristics of the sources of evidence used as eligibility criteria (e.g., years considered, language, and publication status), and provide a rationale	7
Information sources	7	Describe all information sources in the search (e.g., databases with dates of coverage and contact with authors to identify additional sources), as well as the date the most recent search was executed	8
Search	8	Present the full electronic search strategy for at least 1 database, including any limits used, such that it could be repeated	9
Selection of sources of evidence	9	State the process for selecting sources of evidence (i.e., screening and eligibility) included in the scoping review	9–10
Data charting process	10	Describe the methods of charting data from the included sources of evidence (e.g., calibrated forms or forms that have been tested by the team before their use, and whether data charting was done independently or in duplicate) and any processes for obtaining and confirming data from investigators	10
Data items	11	List and define all variables for which data were sought and any assumptions and simplifications made	10
Critical appraisal of individual sources of evidence	12	If done, provide a rationale for conducting a critical appraisal of included sources of evidence; describe the methods used and how this information was used in any data synthesis (if appropriate)	N/A
Synthesis of results	13	Describe the methods of handling and summarizing the data that were charted	10–11
RESULTS			
Selection of sources of evidence	14	Give numbers of sources of evidence screened, assessed for eligibility, and included in the review, with reasons for exclusions at each stage, ideally using a flow diagram	11–12
Characteristics of sources of evidence	15	For each source of evidence, present characteristics for which data were charted and provide the citations	12–14
Critical appraisal within sources of evidence	16	If done, present data on critical appraisal of included sources of evidence (see item 12)	N/A
Results of individual sources of evidence	17	For each included source of evidence, present the relevant data that were charted that relate to the review questions and objectives	Data charting table available upon request
Synthesis of results	18	Summarize and/or present the charting results as they relate to the review questions and objectives	14–23
DISCUSSION			
Summary of evidence	19	Summarize the main results (including an overview of concepts, themes, and types of evidence available), link to the review questions and objectives, and consider the relevance to key groups	24–25
Limitations	20	Discuss the limitations of the scoping review process	26
Conclusions	21	Provide a general interpretation of the results with respect to the review questions and objectives, as well as potential implications and/or next steps	26–27
FUNDING			
Funding	22	Describe sources of funding for the included sources of evidence, as well as sources of funding for the scoping review. Describe the role of the funders of the scoping review	4

### Eligibility criteria

This review focused on forcibly displaced women who were internationally displaced (not internally within the same country) who have then resettled in HICs. Forced displacement occurs when people are forced to leave their home due to ‘persecution, conflict, violence, human rights violations and events seriously disturbing public order’ [23] (p.2). When referring to forced displacement, Australian literature uses the terms refugee [24], asylum-seeking [25], and forced migration [26]. Internationally, people seeking asylum have applied for refugee status under the 1951 Geneva Convention, and refugees are people who have been granted this status [27]. The term ‘migrant’ is widely used in Europe to include any person who has moved from their home (more often permanently rather than temporarily) [28], whereas the term ‘immigrant’ is commonly used in North America [29]. The current review uses the term ‘forced displacement’ or ‘forcibly displaced’, except when referring to specific literature that may use other terms, defined above, to describe forced displacement.

This review defines HICs using the Organisation for Economic Co-operation and Development [OECD]). The OECD defines 38 HICs that must implement policy and practice standards aligned with legal instruments, ‘to achieve the highest sustainable economic growth and employment and a rising standard of living’ [30] (p.1). This means HICs are comparable on indexes of health, safety, education, and gender differences [31]. Most forcibly displaced people come from low-income countries such as Syria, Afghanistan, South Sudan and Myanmar [23], whereas HICs have the most resources for social integration of forcibly displaced people after their arrival [32]. Thus, findings and recommendations will be based on evidence that has transferability to the Australian context and other HICs alike. Articles were excluded if women were resettling in low- and middle-income countries. This is because the health systems and societies of low- and middle-income countries differ markedly from HICs.

Articles that were unclear or vague in terms of defining ‘forced displacement’ were excluded due to ambiguity. Women could identify as any gender (cisgender, transgender, gender-fluid etc.), sexuality, ethnicity, ability or socioeconomic status. Articles that did not disaggregate between women and other genders (e.g., men) were excluded. Articles were included if they considered influences on RDM after arrival in HICs, including before and during displacement. The focus of this scoping review was the influences on RDM after arrival in HICs however, based on the existing evidence base it was understood that influences after arrival were situated within the context of the process of displacement and resettlement, rather than resettlement alone. Therefore, articles that considered influences before and during displacement were not discounted because of their continued relevance and influence on RDM after arrival in HICs. Articles were excluded if they focused on reproductive health outcomes rather than decision-making.

This review included quantitative, qualitative, and mixed-methods study designs and all types of reviews (e.g., narrative, scoping, systematic). Reviews were included in the current scoping review to best capture the range of available evidence related to the research question, and this method has been utilised in existing scoping reviews [33–35]. Only peer-reviewed articles were included in the review; grey literature (i.e., dissertations, organisational reports, policy documents, conference presentations, opinion pieces, commentaries and books) were excluded. Articles were included if they were published between 2012 and 2022 to ensure currency and relevance. The authors’ predominant language is English, which meant only articles published in English were included.

### Search strategy

The search strategy, search terms and eligibility criteria were developed by AD, and refined during discussions with all authors. A search of the literature was conducted by AD to find articles published from 1 January 2012 to 27 April 2022 using EBSCO and included; Academic Search Complete, CINAHL, Communication & Mass Media, E-Journals, Environment, Global Health, Health Policy Reference Centre, Health Source: Nursing/Academic Edition, Historical Abstracts with Full Text, Humanities Source, LGBTQ + Source, MEDLINE, Political Science, Psychology and Behavioural Sciences Collection, APA PsycINFO, Religion and Philosophy Collection, and SocINDEX. Search terms covered three key concepts, 1) reproduction, 2) decision-making, and 3) forcibly displaced. The search terms for ‘reproduction’ and ‘decision-making’ were based on a recent scoping review [16]. Search terms for ‘forcibly displaced’ were developed based on preliminary literature scans. All search terms were checked individually to ensure they produced results; if a term did not produce results it was removed. Table 2 presents the conducted search.

**Table 2:** Search conducted with EBSCO

Concept	Search terms
Reproduction	Reproduct* OR child bear* OR pregnan* OR prolificacy OR procreat* OR conceive OR conception OR produce offspring OR progenerate OR gravid* OR fertili* OR beget OR impregnat* OR gestation OR antenatal OR prenatal OR termination OR abortion OR preventing pregnancy OR acceptable methods of birth control OR birth control OR contracept* OR abstinence OR family planning OR safe family planning OR acceptable methods of family planning OR affordable family planning AND
Decision-making	Decision making OR empowerment OR autonomy OR decision* OR make decision OR decision making process OR choice* OR choose AND
Forcibly displaced	Refuge* OR refugee background OR asylum OR asylum seeking OR migrant OR emigrat* OR immigrant OR entrant OR displace* OR forcibly displaced OR settlement OR settled OR resettlement OR resettled OR new arrival OR newly arrived OR recent arrival OR recently arrived

Three separate searches were conducted, one for each concept (e.g., search 1 = reproduction, search 2 = decision-making, and search 3 = forcibly displaced). All three searches were then combined using the ‘AND’ operator. Searches were set to identify key terms in the titles and abstracts of articles. The reference lists of the included articles were hand-searched for relevant articles based on titles. This was to ensure any additional relevant articles were included in the review.

### Article selection

Title and abstract screening of the articles produced by the search strategy was undertaken by two independent reviewers. Full-text screening was also undertaken by two independent reviewers. Any conflicts at the title and abstract or full-text screening stages were resolved by a third reviewer via discussion of eligibility criteria. AD screened all titles and abstracts, as well as all full-text with either MG, GLH, or HM being the second reviewer.

### Data extraction and synthesis

Data were extracted from each article by one independent reviewer (AD) using a charting table which included author(s), year of publication, publication type, aims/objectives, study design, sampling method, data collection or eligibility criteria, study population (i.e., sample size and characteristics), migration status, country(ies) of origin, host country(ies), key findings and limitations.

The main data items extracted focused on migration status (whether forcibly displaced), the host country (whether high-income) and RDM. It was assumed that if migration status was defined by conflict in the country of origin, humanitarian entry into the host country, refugee or asylum status, or migration from refugee camps, then women were forcibly displaced. Even though both quantitative and qualitative evidence were included in the review, the data extracted from each were key findings relevant to the research question. This meant that numerical statistics were excluded in favour of identifying the influences on forcibly displaced women’s RDM after arrival in HICs, with interpretations of quantitative results from each article.

Whilst data charting was undertaken for this review, a more nuanced approach to analyse the findings related to forcibly displaced women’s RDM was required. Therefore, inductive thematic analysis was employed to capture the complexity and dynamic nature of the influences on RDM among women from a wide array of cultures and countries. Thematic analysis has been utilised to synthesise quantitative, qualitative and review article findings in existing scoping reviews [33, 36]. Thematic analysis was conducted independently by AD using Microsoft Excel. Initially, analysis produced codes that identified single meanings and concepts, which were later collated to form categories and then preliminary themes. Coding was conducted by identifying segments of the data in the ‘key findings’ column of the data extraction table and assigning each segment to a code or multiple codes. Codes were then combined based on their relation to the overall research question. Themes and subthemes were refined using an iterative process that involved moving between written analysis (a draft write up of themes) and a thematic map [37] (p.85). The thematic map was the beginning of the adapted socio-ecological model. The socio-ecological model was initially used to map the themes to identify strengths and gaps in the evidence base in line with a scoping review approach. The model was further developed and refined as the themes were identified within and across socio-ecological levels, as well as across the course of displacement and resettlement. Additionally, to complement thematic analysis, intersectionality theory was employed to enable in-depth exploration of the nuance within forcibly displaced women’s RDM, especially with considerations of marginalisation. Intersectionality examines the connections between individual identities (e.g., sexuality, gender, race, class, ability) and systems of oppression, including, but not limited to, sexism and racism [38, 39].

### Findings

**Fig. 1:**
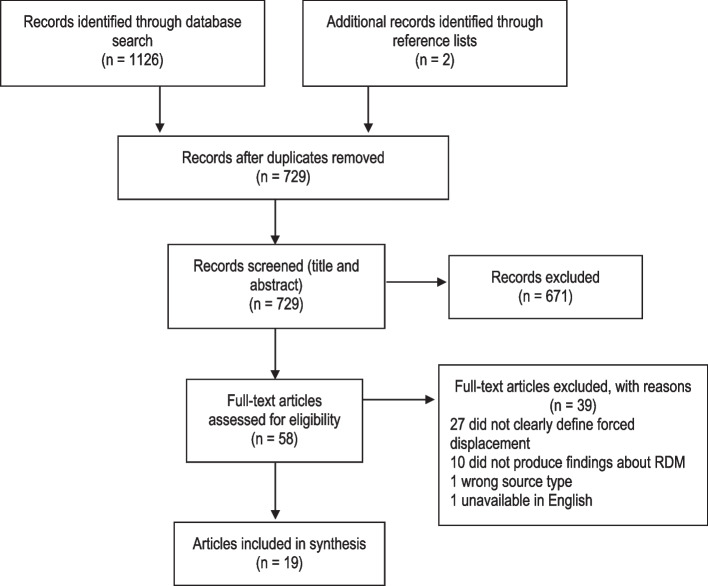
Article selection process

The search yielded 727 articles with duplicates removed and two additional articles were obtained from reference lists. Title and abstract screening of the 729 articles led to the exclusion of 671 articles. Full-text screening was then undertaken on 58 articles that were assessed for eligibility. Thirty-nine articles were subsequently excluded; 27 did not clearly define ‘forced displacement’, 10 did not produce findings about reproductive decision-making, one article was a commentary, and one article was unavailable in English. A total of 19 articles met the eligibility criteria. Figure 1 represents this selection process.

The 19 included articles were published between 2013 and 2022, with sample sizes ranging from 10 to 1,773 people (see Table 3 for article characteristics). Thirteen articles reported on qualitative findings (including two mixed-methods studies that only reported on qualitative data) (13/19 articles, 68.4%) [40, 41], three quantitative (3/19 articles, 15.8%) [42–44], one mixed-methods (1/19 articles, 5.3%) [45], one scoping review (1/19 articles, 5.3%) [46], and one narrative review (1/19 articles, 5.3%) [47]. Of the 17 primary articles, four used convenience sampling (4/17 articles, 23.5%) [42, 43, 45, 48], three used purposive (3/17 articles, 17.6%) [40, 49, 50], four used both purposive and snowball (4/17 articles, 23.5%) [51–54], two used purposive and convenience (2/17 articles, 11.8%) [41, 55], one used informant-purposive and snowball (1/17 articles, 5.9%) [56], one used convenience and snowball (1/17 articles, 5.9%) [57], one used purposive, gatekeeper and snowball (1/17 articles, 5.9%) [58], and one used stratified random sampling (1/17 articles, 5.9%) [44]. Eight of the primary articles conducted interviews (8/17 articles, 47.1%) [43, 48, 50–52, 54–56], three conducted interviews and focus groups (3/17 articles, 17.6%) [53, 57, 58], two conducted focus groups only (2/17 articles, 11.8%) [40, 49], two conducted surveys (2/17 articles, 11.8%) [42, 45], one conducted informal discussions, interviews, collected written journal responses and field notes (1/17 articles, 5.9%) [41], and one conducted secondary analysis of national surveys (1/17 articles, 5.9%) [44].

**Table 3:** Article characteristics

Author(s)	Year	Aims	Study design	Data collection / Eligibility criteria	Sampling method & sample size	Ages and genders in sample	Countries of origin	Host countries	Migration status
Agbemenu et al. [46]	2022	To synthesise current evidence and identify gaps regarding family planning among African immigrant women in the United States	Scoping review	Included articles in English that reported on African immigrant or refugee women Excluded articles that focused on other aspects not explicitly family planning	PRISMA extension for scoping reviews Sample size: 9 studies	18—55 years 100% women	Somalia, the Democratic Republic of Congo, Burundi, Eritrea, Rwanda, Kenya, Sudan, Nigeria, Ethiopia, Ghana, Cameroon	United States	Forcibly displaced defined by civil unrest in home country
Agbemenu et al. [49]	2018	To explore refugee women's reproductive health decision-making, as influenced by their resettlement in the United States	Qualitative	Focus groups (semi-structured)	Purposive sampling Sample size: 30	18 to 55 years 100% women	Somalia, Kenya	United States	Forcibly displaced defined by application for refugee status
Baroudi et al. [42]	2021	To describe the extent to which sexual rights are fulfilled among young migrants in Sweden	Quantitative	Cross-sectional survey	Convenience sampling Sample size: 1773	16 to 29 years 34.9% women 63.1% men 2.0% non-binary	No countries of origin specified Middle East, North Africa, South Asia, Sub-Saharan Africa	Sweden	Forcibly displaced defined by seeking refuge or asylum (not necessarily applying)
Celikkannat & Gungormus [50]	2022	To evaluate refugee women's knowledge and attitudes about family planning	Qualitative	Interviews (in-depth semi-structured)	Purposive sampling Sample size: 30	19 to 45 years 100% women	Syria	Turkey	Forcibly displaced defined by application for refugee status
Cox et al. [55]	2019	To explore couples' communication and decision-making regarding child spacing	Qualitative	Interviews (semi-structured)	Purposive and convenience sampling Sample size: 19	18 to 51 years 47.4% women 52.6% men	Somalia, Djbouti	United States	Forcibly displaced defined by civil unrest in home country
Dhar et al. [48]	2017	To explore attitudes and beliefs about sexual and reproductive health among unmarried, female, resettled refugee women	Qualitative	Interviews (semi-structured)	Convenience sampling Sample size: 14	16 to 20 years 100% women	Nepal	United States	Forcibly displaced defined by being born in refugee camps
Gumus Sekerci & Aydin Yildirim [43]	2020	To explore the knowledge, attitudes and behaviours of refugee women regarding family planning	Quantitative	Interviews (structured)	Convenience sampling Sample size: 389	15 to 49 years 100% women	Syria	Turkey	Forcibly displaced defined by forced migration
Higginbottom et al. [40]	2013	To explore the maternity experiences of Sudanese women resettled in Canada	Mixed-methods (only reported on qualitative data)	Focus groups	Purposive sampling Sample size: 12	25 to 45 years 100% women	Sudan	Canada	Forcibly displaced defined by civil unrest in home country
Mantovani & Thomas [51]	2014	To explore the experiences of young refugee women regarding pregnancy decision-making	Qualitative	Interviews (in-depth unstructured)	Purposive and snowball sampling Sample size: 15	16 to 19 years 100% women	No countries of origin specified South West, West and East Africa	United Kingdom	Forcibly displaced defined by seeking refuge or asylum (not necessarily applying)
McMichael [41]	2013	To examine the ways young refugee women negotiate teen pregnancy and early motherhood whilst managing the challenges of resettlement	Mixed-methods (only reported on qualitative data)	Informal discussions, interviews, written journal responses and field notes	Purposive and convenience sampling Sample size: 120	11 to 19 years 45.8% women 54.2% men	Sudan, Ethiopia, Liberia, Uganda, Burundi, Iraq, Afghanistan, Iran, Kuwait, Bosnia, Croatia, Burma	Australia	Forcibly displaced defined by application for refugee status
Mengesha et al. [56]	2017	To examine the perspectives and practices of healthcare professionals regarding their provision of sexual and reproductive healthcare for refugee and migrant women	Qualitative	Interviews (semi-structured)	Informant-purposive and snowball sampling Sample size: 21	32 to 70 years 100% women	No countries of origin specified	Australia	Used the phrase 'recent refugee and migrant women'
Metusela et al. [57]	2017	To examine experiences of sexual and reproductive healthcare among non-English-speaking migrant and refugee women	Qualitative	Interviews and focus groups	Convenience and snowball sampling Sample size: 169	18 to 70 years 100% women	Afghanistan, Iraq, Somalia, South Sudan, Sudan, India, Sri Lanka, South America	Australia and Canada	Used the phrase 'non-English-speaking migrant and refugee women'
Ngum Chi Watts et al. [52]	2014	To explore the contraception knowledge, attitudes and beliefs of African refugee women in Melbourne	Qualitative	Interviews (in-depth structured)	Purposive and snowball sampling Sampling size: 16	17 to 30 years 93.6% women 6.4% men	Sudan, Liberia, Ethiopia, Burundi, Sierra Leone	Australia	Forcibly displaced defined by application for refugee status
Ngum Chi Watts et al. [58]	2015	To examine contraception awareness and use among African refugee women in Melbourne	Qualitative	Interviews (in-depth) and focus groups	Purposive, gatekeeper and snowball sampling Sample size: 16	17 to 30 years 100% women	Sudan, Liberia, Ethiopia, Burundi, Sierra Leone	Australia	Forcibly displaced defined by application for refugee status
Saint Felix [47]	2019	To outline and discuss the stories of young women detained at a Texas immigration detention centre who were pregnany, undocumented immigrants that were denied access to abortion	Narrative review	No eligibility criteria described	Not applicable	No sample	Mexico	United States	Forcibly displaced defined by being detained at an immigration detention centre
Soin et al. [53]	2020	To examine the factors influencing family planning choices of resettled refugee women in the United States	Qualitative	Interviews (in-depth) and focus groups	Purposive and snowball sampling Sample size: 32	19 to 73 years 100% women	Nepal, Burma, Iraq	United States	Forcibly displaced defined by seeking refuge or asylum (not necessarily applying)
Vaisanen et al. [44]	2018	To compare the relationship between having an abortion and current contraceptive use among Kurdish, Somali and Russian migrant women and the general population in Finland	Quantitative	Secondary analysis of national surveys	Stratified random sampling Sample size: 1000	18 to 64 years 100% women	Russia (or the former Soviet Union), Iran, Iraq, Turkey, Somalia	Finland	Recent immigrant women
Verran et al. [54]	2015	To examine the experiences and decisions about family planning among Chinese women seeking asylum in the United Kingdom	Qualitative	Interviews (semi-structured)	Purposive and snowball sampling Sample size: 10	26 to 41 years 100% women	Mainland China	United Kingdom	Forcibly displaced defined by seeking refuge or asylum (not necessarily applying)
Zimmerman & Beam [45]	2020	To examine the information-seeking patterns, needs and barriers among refugee women regarding their health	Mixed-methods	Cross-sectional survey	Convenience sampling Sample size: 85	18 to 68 years 100% women	No countries of origin specified Central America, Africa	United States	Forcibly displaced defined by application for refugee status

Fifteen articles included only women (15/19 articles, 78.9%) [40, 43–54, 56, 57], three included both men and women (3/19 articles, 15.8%) [41, 55, 58], and one included women, men and non-binary participants (1/19 articles, 5.3%) [42]. Participant ages ranged from 11 to 73 years, with seven articles including participants under the age of 18 (7/19 articles, 36.8%) [41–43, 48, 51, 52, 58]. Seven of the studies were conducted in the United States (7/19 articles, 36.8%) [45–49, 53, 55], four in Australia (4/19 articles, 21.1%) [41, 52, 56, 58], two in the United Kingdom (2/19 articles, 10.5%) [51, 54], two in Turkey (2/19 articles, 10.5%) [43, 50], one in Canada (1/19 articles, 5.3%) [40], one in Australia and Canada (1/19 articles, 5.3%) [57], one in Sweden (1/19 articles, 5.3%) [42], and one in Finland (1/19 articles, 5.3%) [44].

Women’s countries of origin varied. Seven articles included women from African countries only, including Somalia, Kenya, Sudan, South Sudan, Sierra Leone, Burundi, and Ethiopia (7/19 articles, 36.8%) [40, 46, 49, 51, 52, 55, 58]. Two articles included women from Syria (2/19 articles, 10.5%) [43, 50], two included women from Nepal (2/19 articles, 10.5%) [48, 53], one focused on women from Mexico (1/19 articles, 5.3%) [47], and one included women from mainland China (1/19 articles, 5.3%) [54]. Four articles included women from countries across continents, including Uganda, Iraq, Bosnia, India, Myanmar, Afghanistan, Sri Lanka, and Russia (4/19 articles, 21.1%) [41, 44, 53, 57]. Three articles did not specify women’s countries of origin (3/19 articles, 15.8%) [42, 45, 56].

Twelve articles only included forcibly displaced populations (12/19 articles, 63.2%). Of these, five defined forced displacement by application for refugee status (5/12 articles, 41.7%) [41, 49, 50, 52, 58], two by civil unrest in the home country (2/12 articles, 16.7%) [40, 55], and two by seeking refuge or asylum (not necessarily applying) (2/12 articles, 16.7%) [53, 54]. The narrative review included women detained at an immigration detention centre (1/12 articles, 8.3%) [47], one study defined ‘forced migration’ (1/12 articles, 8.3%) [43], and one included women who were born in refugee camps (1/12 articles, 8.3%) [48]. Five articles disaggregated between forced and voluntary migration (5/19 articles, 26.3%) [42, 44–46, 51]. One article used the phrase ‘recent refugee and migrant women’ (1/19 articles, 5.3%) [56], and another used ‘non-English speaking migrant and refugee women’ (1/19 articles, 5.3%) [57].

### Synthesis of results

Three main contexts apparent in the literature demonstrated the influences across the course of displacement: *Before displacement*; *during displacement*; and *after arrival*. Whilst the focus was on the influences after arrival, it became apparent throughout the analysis that influences before and during displacement played a crucial role regarding RDM after women were resettling in HICs. Many of the themes within these contexts identified are embedded within culture and religion. However, the initial influences (before displacement) of religious beliefs on women’s RDM were especially notable.

### Before displacement

Eleven studies included influences on women’s RDM before displacement, in the country of origin [43, 44, 46, 49–55, 57]. This theme demonstrated that many influences on women’s RDM before displacement, pressured women towards having children and away from using contraception. Many of the influences limited women’s reproductive autonomy, and included *conflict*; *religious beliefs*; *socio-cultural gendered expectations*; and *external control over reproductive autonomy*.

#### Conflict

Conflict, such as civil war, in women’s home countries, pressured women to reproduce, especially young women. Three articles identified conflict as limiting women’s RDM [49, 51, 53]. One article that included women from Somalia and Kenya found women expressed a need to have many children, and a taboo against being childless [49]. For example, one woman reported, ‘anything can happen’ and this phrase was contextualised by experiences of civil war, when death could happen at any time [49] (p.3359). This meant women would not even speak the phrase, ‘I will not have another baby’ because it was ‘forbidden’, and subsequently, women felt compelled to have children, especially if they were young [49] (p.3359). Another article reported young African women experienced ethnic conflicts before displacement, when many conceived as a result of rape, ‘…when the rebels invaded our area they capture[d] so many young girls and I was a victim of that’ [51] (p.74). Conflict in women’s home countries (Nepal, Myanmar and Iraq) reportedly limited access to resources and education about reproductive health, which restricted women’s decision-making power regarding family planning and contraception [53].

#### Religious beliefs

Religious beliefs controlled women’s fertility, timing of pregnancies, number of children, and contraceptive use. Five articles focusing on women from African countries and Syria found women believed Allah or God controlled their fertility and timing of pregnancies [43, 46, 50, 55]. One article reported that these beliefs among Syrian women resulted in consistent unplanned pregnancies [50]. Two articles reported that for African women, child spacing, and contraceptive use was determined by the teachings of Islam [49, 55]. For example, breastfeeding to space pregnancies was the only accepted form of birth control according to Islam [55]. Two articles reported that family planning and contraception were explicitly a sin and forbidden due to women’s religious beliefs [50, 57]. It was also suggested in one article that Kurdish and Somali women had negative attitudes towards using contraception that were partly based on religion [44].

#### Socio-cultural gendered expectations

Socio-cultural gendered expectations which promote motherhood influenced women’s decisions to have children, the number of children and whether to use contraception. Five articles reported that women felt pressured by socio-cultural norms and gendered expectations to have children in their home countries [43, 50, 54, 57, 58]. One article with young African mothers found socialisation towards marriage and childbearing led to more unplanned pregnancies [58]. However, this article also reported mothers were considered ‘high status’, which meant women did not mind having unplanned pregnancies because they valued motherhood [58]. Another article reported that Syrian women believed reproductive decisions should be made in collaboration with their partner, yet often felt pressured by their husband to have many children [50].

Four articles reported that women also felt pressured to have male children which led to more pregnancies [43, 50, 54, 57]. Patriarchal beliefs that ascribed strict gender roles to women were found to result in pressure on women to become pregnant in order to have male children [50, 57]. For example, one article reported that women seeking asylum from China felt pressured by family members and friends to become pregnant again if they did not have a male child [54].

#### External control over reproductive autonomy

External control over RDM limited women’s reproductive autonomy via cultural practices and policies. Four articles found that force (ensuring a particular decision is made) and restriction (removing conditions to make autonomous decisions) were exerted through cultural practices [50, 53, 57] and restrictive policies [54] entrenched in gender inequality. One article reported that Syrian women felt forced into pregnancy because if they did not have children this often led to polygamy, which meant women would have children, even if they did not want to, to prevent their husband from bringing more women/wives home [50]. Another article reported that the practice of early marriage restricted young women from accessing information about contraception, which led to unwanted pregnancy [53].

One article described how ‘further pregnancies were prevented through the mandatory use of long-term contraception’ based on state policy that controlled the number and spacing of children, which meant women felt that contraception and abortion were ‘forced upon them’ in China [54] (p.124). These practices and policies were externally exerted over women’s RDM by husbands, family members [50, 53, 57], and the state [54].

### During displacement

Two articles included influences on women’s RDM during displacement [47, 53]. These influences occurred whilst women were in refugee camps [53] and immigration detention [47]. These articles identified paternalism and access to education as influencing women’s RDM. Paternalism, ‘appears to protect women’ yet is often ‘unconcerned with women’s autonomy, especially in abortion policies’ [47] (p.416). One article reported that anti-abortion ideologies enabled policies which cultivated distrust in women’s ability to make their own reproductive decisions, and allowed immigration officials to deny women’s right to access safe abortion whilst in immigration detention [47].

In contrast to a paternalistic approach, providing access to education can support women with their knowledge about reproductive options and decision-making. For example, one article reported that women in refugee camps attended family planning workshops, which meant they learnt about birth control and could access free family planning [53]. This led women to consider limiting family size, which reportedly increased their economic opportunities because of fewer caring responsibilities [53].

### After arrival

Eighteen articles focused on the influences on women’s RDM after arrival [40–46, 48–58], whereby the influences occurred after women arrived in a HIC and began the resettlement process (i.e., released from immigration detention). Four influences were identified: *pressure, restriction, coercion*; *knowledge and misconceptions*; *patriarchal power dynamics*; and *seeking empowerment*.

#### Pressure, restriction, coercion

Pressure, restriction, and coercion influenced women’s RDM by pressuring women to have children, yet in some cases to have an abortion. Women’s access to services and contraception was restricted, and women experienced coercion toward utilising contraception and abortion in HICs. Eight articles reported women experienced pressure, restriction and coercion from partners and parents [41, 43, 46, 50, 57, 58], healthcare professionals [51] and the healthcare system [56].

One article with Syrian women who had resettled in Turkey reported that pressure to have children was compounded by women’s economic circumstances and language barriers [50], which then worked to ‘neutralise’ the positive impacts of migrating from a low-income to a HIC where services are more ‘readily available’ [50] (p.282). Another article found if women wanted to use contraception their partners believed this meant women also wanted to ‘cheat’ and condoms were viewed as a ‘sign of distrust’ by women’s partners [58] (p.12–13). ‘Males would threaten to leave a relationship if the woman insisted, he use a barrier method’ [58] (p.13). As a result, women tended to ‘risk’ unplanned pregnancy.

Two articles reported women’s access to family planning services and contraception were restricted by their partners [43, 57]. Moreover, one article reported that young African women resettling in Australia, were pressured not to use contraception by their parents because they believed contraception would lead to sexual promiscuity [58]. However, it was reported that mothers would then encourage their unmarried, pregnant daughters to have an abortion to avoid perceived shame on the family [51]. One article reported that young refugee women were found to experience a lack of social support after arriving in the United Kingdom, which meant their parents had a profound influence on their RDM [51].

As well as parents and partners, one article reported that healthcare professionals in the United Kingdom held ‘stereotyped judgements’ about young refugee women, and these judgments meant professionals encouraged women to have an abortion and did not ensure their right to access alternative options [51] (p.76). Another article reported that healthcare professionals ‘believed that [Sexual and Reproductive Health] care in Australia is inclined towards STI screening and contraception with little attention to sexual functioning and relationship areas’, which was discussed as an institutional level barrier that restricted women’s ‘access and utilisation’ of SRH care [56] (p.14).

#### Knowledge and misconceptions

Knowledge and misconceptions influenced women’s choice to use mechanisms that control fertility, especially contraception. Nine articles reported that women, from Sudan, Ethiopia, China, Sri Lanka, Afghanistan, Iraq, and Nepal, held socio-cultural beliefs which led to misconceptions about reproductive decisions in HICs [42–44, 48–50, 52, 54, 57]. However, one article found women resettling in the United States, deemed birth control and family planning the most important health information needs for refugee women [45].

Two articles reported that women’s knowledge about reproductive health and access to services increased after arriving in Sweden and the United States [42, 48]. Women were ‘aware of the male condom’ and knew where to access them [48](p.792), also more women than men knew where to access contraceptives [42]. Moreover, one article reported that Syrian women resettling in Turkey knew the minimum time needed between pregnancies and at least two methods of contraception [43]. However, in the United States, refugee women were found to avoid discussions with healthcare professionals about hormonal birth control because they believed the side effects (mood swings) would negatively impact the relationship with their husband [49].

Misconceptions were found to discourage women from using contraception [52, 54]. For example, one article reported that African women resettling in Australia believed contraception would cause irreversible damage to their fertility or even cancer, which led women to take ‘breaks’ in their contraceptive use [52] (p.2137). These women felt a ‘…loss of control’ over their reproductive decisions and ‘discouraged’ to use contraception based on misconceptions [52] (p.2137). Another article found women who sought asylum from China preferred the non-hormonal IUD in the United Kingdom because this was the only contraceptive method used in their home country [54]. These women had concerns about the side effects related to hormonal methods of contraception, and this was compounded by their experiences of ‘language barriers’ and ‘cultural misunderstandings’ during health service interactions after arrival in the United Kingdom [54] (p.124). In Finland, one article reported that Kurdish women in contact with health services after an abortion led to an increased likelihood of using contraception [44]. Although, this was not the case for Somali women who were less likely to commence contraception [44].

Three articles found that women attributed misconceptions to a lack of support from their communities [52], and restricted access to health services by their husbands and family [50, 57].

#### Patriarchal power dynamics

Patriarchal power dynamics influenced women’s decision to use methods to control fertility, as well as spacing and number of pregnancies. Five articles found forcibly displaced women experienced patriarchal power dynamics that influenced their RDM after arrival in HICs [48–50, 55, 56]. Two of these articles reported that women’s husbands did not support contraceptive use [49, 50]. One article focusing on African women resettling in the United States found women wanted adequate spacing between pregnancies, yet men’s desire to have many children often prevailed [49]. It was also reported that healthcare professionals expressed concerns about the ‘patriarchal gender structure that gave husbands most of the power over their wife’s sexuality and her ability to access contraception care’, leading the authors to suggest women were experiencing reproductive coercion [56] (p.10). Another article found unmarried women were not allowed to access reproductive health services or information, and women believed their male partner would need to provide consent for an abortion [48]. However, another article reported that couples resettling in the United States shared that they openly discussed their opinions about using contraception and women were said to have the final decision [55].

#### Seeking empowerment

Seeking empowerment influenced women’s contraceptive use because women resisted practices that oppressed their reproductive autonomy. Five articles found women sought empowerment from restrictive cultural practices after arrival in HICs [40, 49, 53, 57, 58]. One article reported that men’s power over reproductive decisions led Sudanese women to use contraception secretly [40]. This was viewed by women as enacting ‘personal agency’, and an ‘empowering decision’ for women to ‘gain some control over their reproductive decisions’, which lead to having fewer children after arriving in Canada [40] (p.5). Another two articles also found women felt empowered using contraception [53, 58]. This included women resettling in Australia who reported using contraception provided ‘…opportunity to manage fertility and to plan their families’ [58] (p.13). It was also reported that women felt empowered to reject ‘…oppressive customs from their past’ through the increased acknowledgement of women’s rights and access to reproductive health information after arrival in the United States [53] (p.73). In another article, African women resettling in the United States justified their right to use contraception because of their greater caring responsibility [49]. It was also found that despite the insistent pressure for women to reproduce, women showed interest in receiving information about contraception, negotiating sex within marital relationships, and wanted their husbands to receive sexual and reproductive health education [57].

### Mapping the themes: Socio-ecological model

**Fig. 2:**
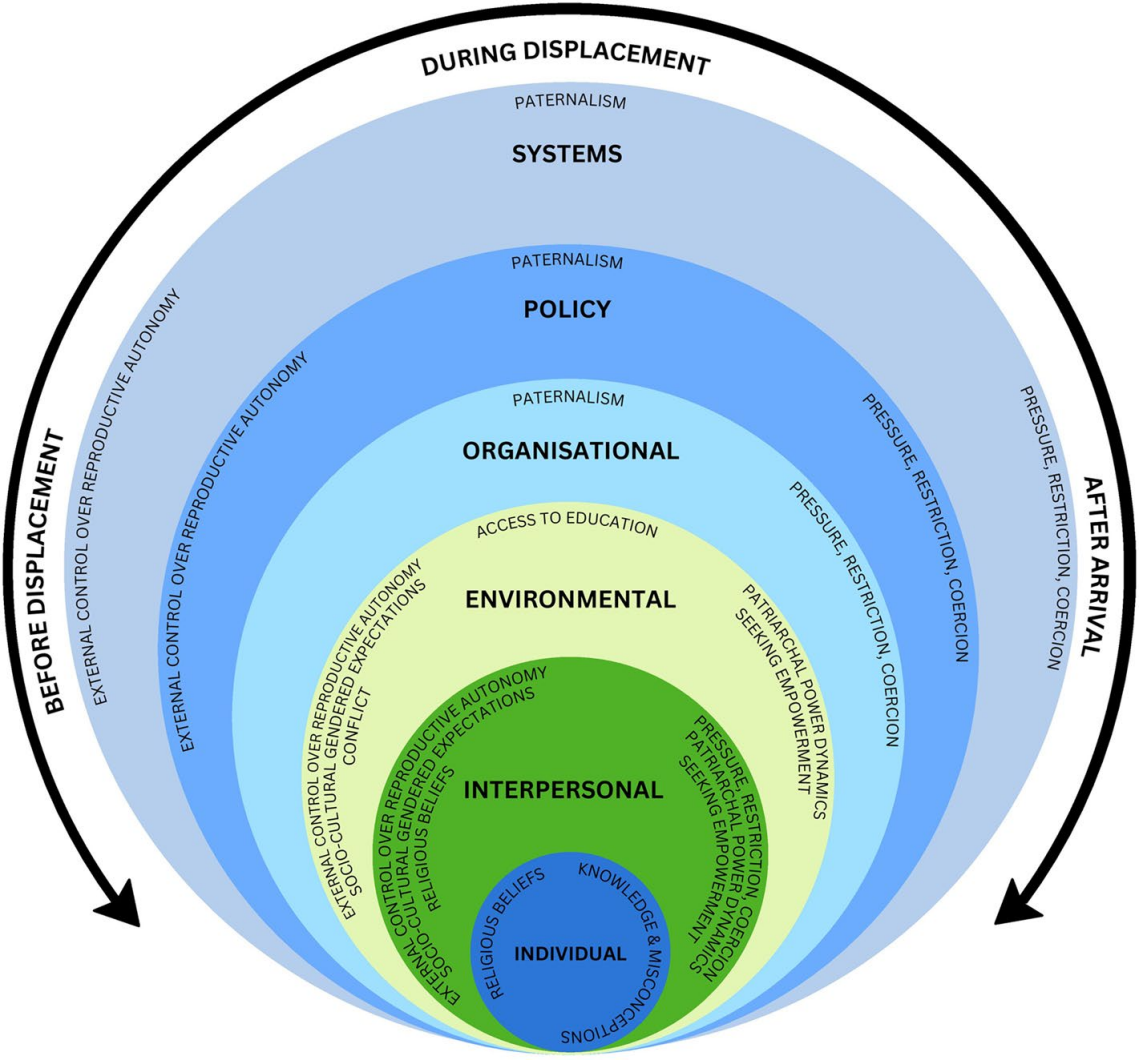
Adapted temporal socio-ecological model*.* Note. Adapted from Understanding health (4th ed. p.98), by H. Keleher and C. MacDougall, 2016, Australia: Oxford. Copyright 2016 by Copyright Agency Limited [59]. However, during analysis it became apparent that the fifth level should be referred to as ‘policy’ rather than ‘societal’. This is because a more specific policy level best reflected the findings from the analysis of this review. To ensure intersectionality informed interpretation of the findings, a systems level was also added as the outermost level of the model. The temporal element was adapted to the model to demonstrate where along a continuum of forced displacement the influences on reproductive decision-making were most apparent based on the reviewed literature.

Based on the themes derived from the evidence base, it is clear that individual, interpersonal, environmental, organisational, and policy-level influences on women’s RDM interacted across the course of displacement and resettlement (see Fig. 2). The temporal element was adapted to the model to demonstrate where along the continuum of forced displacement and resettlement the influences on women’s RDM were most apparent based on the reviewed literature. Further, intersectionality theory was applied to view the systems of oppression related to individual identities and lived experiences that shape the influences on women’s RDM. This led to a systems level being added as the outermost layer of the model.

### Individual

The findings demonstrate that from an individual level women internalised stereotypes about their identities and experiences which led to misconceptions that then discouraged women from making autonomous reproductive decisions in HICs. This restricted women’s RDM which meant they were more susceptible to reproductive coercion. For example, negative stereotyped judgements about young refugee women held by healthcare professionals led these professionals to enact reproductive coercion, especially towards abortion [51]. This suggests healthcare professionals, whether deliberately or inadvertently, are restricting young forcibly displaced women’s reproductive autonomy, and thus, their reproductive rights in HICs. With an intersectional lens, it can be understood that even individual-level influences (e.g., religious beliefs, age, migration status) are interpreted and treated by society to influence women’s RDM, yet remain out of the control of women themselves.

### Interpersonal

Interpersonal influences on women’s RDM involved pressure to reproduce, yet also women’s resistance to this pressure. In HICs, women continued to experience *socio-cultural gendered expectations* pressuring motherhood. *Patriarchal power dynamics* often meant husbands would control women’s RDM. Although in one article that interviewed both men and women (albeit separately) who were in a relationship, reproductive decisions were said to ultimately be up to the woman who had the final decision [55]. This contrasted with other articles that only included women, where women shared they felt pressured by their partners to have children [46, 50, 58]. This finding highlights that *patriarchal power dynamics* not only influence women’s RDM, but also what women share with researchers and healthcare professionals about their RDM whilst knowing that their partner is participating in the conversation.

However, the current review also found women demonstrated resistance by *seeking empowerment* from *socio-cultural gendered expectations* and *patriarchal power dynamics* after arrival in HICs. This finding is supported by recent evidence that emphasised women’s acts of resistance that ‘remake culturally normative models of womanhood’ rather than simplified explanations attributed to ‘cultural values’ or ‘traditional beliefs’ [20] (p.14). The former centres women’s agency rather than the external forces that restrict women’s RDM in HICs.

### Environmental

Environmental influences often limited women’s reproductive autonomy before displacement. *Conflict, religious beliefs* and *socio-cultural gendered expectations* led women to have unplanned or unwanted pregnancies. These influences before displacement were especially detrimental for young women who continued to experience *pressure, restriction, coercion* after arrival, despite the promotion of gender equality in HICs. However, some women sought empowerment from traditions that were no longer supported by the socio-cultural context of their home countries. The reviewed literature suggests women sought empowerment as a response to patriarchal gendered expectations of motherhood that they believed were obsolete in HICs [40, 49].

### Organisational

It is noteworthy that almost all included studies reported findings from forcibly displaced women themselves, rather than healthcare professionals. This is worth highlighting here because although healthcare professionals give valuable insight about the influences on forcibly displaced women’s RDM, healthcare professionals and systems also enact organisational influence that limits women’s reproductive autonomy [51, 56, 57]. However, organisational influences were one of the least considered among the reviewed literature.

### Policy

Policy-level influences were also limited among the reviewed literature. HICs, although considered relatively progressive, still have punitive policies that perpetuate gender inequality and exclude women’s intersectional experiences. After arrival, *pressure, restriction, coercion* have political influence that limited forcibly displaced women’s reproductive autonomy. Health policy in HICs seemed to neglect the range of facets that encompass women’s reproductive health. Instead, it was reported that healthcare focused on contraception and sexually transmitted infections, which then limited the range of options and decision-making power given to women by healthcare professionals [56].

### Systems

The reviewed literature demonstrated that young, forcibly displaced women who were also pregnant and unmarried were more often treated with *pressure, restriction, coercion* over their reproductive autonomy after their arrival in HICs. This treatment was exerted by partners, parents, healthcare professionals and systems [41, 51, 58], based on negative stereotypes of young forcibly displaced women. Across the course of displacement, *external control over reproductive autonomy*, *paternalism,* and *pressure, restriction, coercion* were all systemic influences of reproductive oppression that substantially impacted young, forcibly displaced women, especially if they were pregnant and unmarried. Young women experienced reproductive pressure towards abortion, restriction from alternative options, and coercion via denial of their rights, after arrival in HICs [51, 56, 57]. Young women frequently experienced limitations on their reproductive autonomy, and even reproductive coercion from a systemic level that interacted with individual-level influences (i.e., *knowledge and misconceptions*), and enabled reproductive *pressure, restriction, coercion* across interpersonal, environmental, organisational and policy levels after arrival in HICs. The literature included in this review was limited when considering paternalism as an influence on forcibly displaced women’s RDM. However, the findings from this review suggest that paternalism is commonly exerted over young, pregnant, unmarried, forcibly displaced women across socio-ecological levels.

## Discussion

This scoping review explored the influences on reproductive decision-making (RDM) among forcibly displaced women resettling in high-income countries (HICs). The findings from this review show that influences consistently restricted reproductive autonomy among forcibly displaced women from before to during displacement, and after arrival in HICs. The reviewed literature indicates women were often pressured to reproduce, especially before displacement yet this was also apparent after arrival in HICs. After arrival, women’s access to contraception and services was commonly restricted by partners and parents. Further, young women were particularly susceptible to reproductive coercion from parents, healthcare professionals and systems in HICs. However, there was also evidence of forcibly displaced women’s resistance to reproductive pressure and coercion in HICs.

A key outcome of this review is an adapted socio-ecological model that frames the influences on forcibly displaced women’s RDM. Previous studies have interpreted their findings with a socio-ecological model to enable a better understanding of the reproductive health of refugee and migrant women across individual, interpersonal, environmental, organisational, and policy levels [11, 19]. However, there is yet to be an integration of socio-ecological and temporal elements on a model that depicts the influences on forcibly displaced women’s RDM. Before displacement, *External control over reproductive autonomy* reaches from interpersonal through to the systems level when influencing women’s RDM. During displacement, *Paternalism* is enacted across organisational, policy and systems levels to influence women’s RDM. Finally, and most important to this review, after arrival in HICs, *Pressure, restriction, coercion* influence women’s RDM across interpersonal, organisational, policy and systems levels alongside *Patriarchal power dynamics* at interpersonal and environmental levels. After arrival, *Patriarchal power dynamics* were met with *Seeking empowerment* at the interpersonal and environmental levels, which represented women’s resistance to reproductive oppression in HICs where the socio-cultural context was more likely to support gender equality. However, *Pressure, restriction, coercion* continue to oppress women’s RDM, suggesting that even with a socio-cultural context supporting gender equality, organisations, policy and systems remain oppressive towards women’s RDM at the intersection of gender and migration status.

The adapted socio-ecological model demonstrates how influences on forcibly displaced women’s RDM change over the course of displacement and resettlement. In line with the existing evidence base, the current review also demonstrates that there are both positive and negative influences on women’s RDM after arrival in HICs however, overall *Patriarchal power dynamics* and *Pressure, restriction, coercion* strongly influence forcibly displaced women’s RDM. The findings from this review draw attention to where these influences are situated and interact within and across socio-ecological levels. This understanding in conjunction with the existing evidence base which suggests forcibly displaced women’s likely decline in health after arrival in HICs, poorer reproductive health outcomes, and barriers to access health services, as well as increased risk of experiencing reproductive coercion, highlight the need for researchers, healthcare professionals and programs, as well as policymakers to respond to the needs of forcibly displaced women. The continued pressure, restriction and coercion influencing RDM after arrival in HICs implicates any response to centre forcibly displaced women’s right to health, and thus, to make autonomous and empowered reproductive decisions. Conceptualising the influences on women’s RDM after arrival in HICs also helps to illuminate gaps or limitations in the breadth of current research on the topic. For example, there is a good deal of literature about influences more proximal to the women, such as influences at the individual, interpersonal, and environmental levels. There is comparatively less literature discussing influences at more distal levels, in particular the organisational level, as well as the systems and policy levels. This reveals the need for further research exploring influences at these levels.

### Implications

This review informs future research, practice and policy, to achieve the 2030 Agenda for Sustainable Development [5]. The goals that work to ensure health for all, and gender equality are relevant to the findings of the current review. Moreover, the OECD Gender Recommendations aim to foster gender equality through inclusive and comprehensive policies in HICs [60, 61], and work to meet SDG 5, towards achieving gender equality by empowering all women and girls [62]. Recent and current global events, such as COVID-19 and the climate crisis are known to worsen inequity experienced by displaced women [63]. HICs are often perceived as progressive and making changes for social equity. However, this review shows that forcibly displaced women consistently experience limits on their reproductive autonomy after arrival in HICs. Thus, empowerment is key to upholding women’s rights and achieving gender equality. Importantly, women’s right to make autonomous reproductive decisions is crucial to attain the highest standard of health for all.

### Strengths and limitations

This scoping review contributes a way of conceptualising this topic not previously explored and demonstrates influences that restrict and empower women’s reproductive decisions across the course of displacement, as well as socio-ecological levels. This also enabled critical understanding and discussion regarding knowledge strengths and gaps. Another strength of this review is that findings were viewed through an intersectional lens which improved understanding of systemic oppression experienced by young forcibly displaced women. The reviewed literature also included women from a wide array of cultures and countries, who had resettled in HICs across Northern Europe, North America, and Australia, thus improving transferability of the findings.

As with any review there were limitations. Firstly, only articles published in English were included. Many HICs’ dominant language is not English, and this would have limited the scope. Also, some of the studies in this review combined populations that migrated voluntarily and forcibly. Even though most articles disaggregated their populations, interpretation of the findings was at times challenging when determining which findings applied only to forcibly displaced women. Another limitation is that only peer-reviewed articles were included, which also limited the scope as grey literature was not searched. There was also a large variation among study methodologies and no quality assessment conducted, which is an optional inclusion for a scoping review.

## Conclusions

This scoping review highlights the complexity and nuance within forcibly displaced women’s experiences across a wide array of cultures and countries. It also demonstrates the similarities in women’s experiences because many of the influences restricted forcibly displaced women’s reproductive autonomy. This is important to link to women’s rights, and especially the right to make autonomous reproductive decisions.

Based on the findings of this review, it is recommended that future research focuses on organisational- and policy-level influences on forcibly displaced women’s RDM after arrival in HICs. This may include a further scoping review and/or a systematic review that focuses on the experiences of young forcibly displaced women to develop specific guidance for healthcare professionals and health policies that work to empower women to make autonomous reproductive decisions in HICs. For practice, it is recommended that programs address, and respond to, the pressure, restriction, and coercion of women’s RDM across socio-ecological levels (i.e., interpersonal, organisational, policy, and systems). The findings suggest that healthcare professionals require training to prevent pressure, restriction and coercion of women’s reproductive autonomy in HICs. In particular, there is a need to ensure discussions with forcibly displaced women regarding reproductive health and decision-making are appropriate and strengths-based, emphasising women’s resistance to reproductive oppression, as well as empowering their choice to make autonomous reproductive decisions, whilst recognising the socio-cultural-political-systemic influences that mediate these discussions. Also, policy must take an intersectional approach that considers the impact of systemic oppression that reaches from individual through to policy-level influences on forcibly displaced women’s RDM. To do so, there must be recognition of the impact of policy on forcibly displaced women especially at the intersection of their gender and migration status. The displacement and resettlement process undoubtedly has an influence on women’s RDM after arrival in HICs, and policies that partner and empower forcibly displaced women by centring their experiences of reproductive oppression are needed. The adapted model contributes a new way to understand the influences on forcibly displaced women’s RDM, and can inform further research, practice, and policymaking for health promotion. In sum, there are individual, interpersonal, environmental, organisational, policy, and systems-level influences on forcibly displaced women’s RDM when resettling in HICs that continue to limit their reproductive autonomy.

## Data Availability

The datasets used and/or analysed during the current study are available from the corresponding author on reasonable request.

## Abbreviations

RDM: Reproductive decision-making
HIC: High-income country
